# Defective TiO_2_ with high photoconductive gain for efficient and stable planar heterojunction perovskite solar cells

**DOI:** 10.1038/ncomms12446

**Published:** 2016-08-18

**Authors:** Yanbo Li, Jason K. Cooper, Wenjun Liu, Carolin M. Sutter-Fella, Matin Amani, Jeffrey W. Beeman, Ali Javey, Joel W. Ager, Yi Liu, Francesca M. Toma, Ian D. Sharp

**Affiliations:** 1 Joint Center for Artificial Photosynthesis, Lawrence Berkeley National Laboratory, 1 Cyclotron Road, Berkeley, California 94720, USA; 2 Chemical Sciences Division, Lawrence Berkeley National Laboratory, 1 Cyclotron Road, Berkeley, California 94720, USA; 3 Materials Sciences Division, Lawrence Berkeley National Laboratory, 1 Cyclotron Road, Berkeley, California 94720, USA; 4 Electrical Engineering and Computer Sciences, University of California, Berkeley, California 94720, USA; 5 Materials Science and Engineering, University of California, Berkeley, California 94720, USA; 6 Molecular Foundry, Lawrence Berkeley National Laboratory, 1 Cyclotron Road, Berkeley, California 94720, USA

## Abstract

Formation of planar heterojunction perovskite solar cells exhibiting both high efficiency and stability under continuous operation remains a challenge. Here, we show this can be achieved by using a defective TiO_2_ thin film as the electron transport layer. TiO_2_ layers with native defects are deposited by electron beam evaporation in an oxygen-deficient environment. Deep-level hole traps are introduced in the TiO_2_ layers and contribute to a high photoconductive gain and reduced photocatalytic activity. The high photoconductivity of the TiO_2_ electron transport layer leads to improved efficiency for the fabricated planar devices. A maximum power conversion efficiency of 19.0% and an average PCE of 17.5% are achieved. In addition, the reduced photocatalytic activity of the TiO_2_ layer leads to enhanced long-term stability for the planar devices. Under continuous operation near the maximum power point, an efficiency of over 15.4% is demonstrated for 100 h.

## Abstract (web summary)

Perovskite-based photovoltaics have already reached high efficiency levels but their stability under operating conditions remains a challenge. Here, Li *et al.* use defective TiO_2_ with reduced photocatalytic efficiency to increase the stability of high efficiency solar cells under illumination

## Graphical abstract

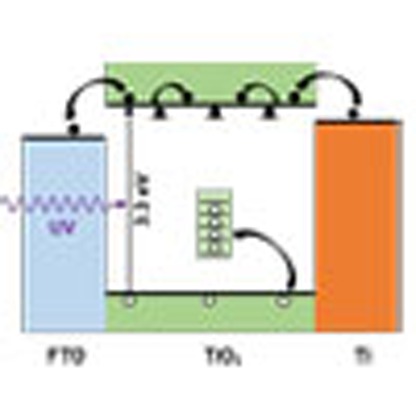

Organo-lead halide perovskites show significant potential as candidates for next-generation thin-film photovoltaics, with demonstrated solar cell efficiencies increasing from 3.8% in 2009 to over 20% in 2015 (refs 1, 2). This unprecedented progress is made possible by the outstanding material properties of perovskites, including direct bandgaps suitable for capturing the solar spectrum, long and balanced charge carrier diffusion lengths, and high defect tolerance^3^,^4^,^5^. Despite the ultra-long carrier diffusion lengths in these materials^4^, progress in the field has been dominated by perovskite solar cells (PSCs) that use mesoscopic TiO_2_ electron transport layers (ETLs) derived from the dye-sensitized solar cell community. For example, the first PSC with a power conversion efficiency (PCE) above 15% was demonstrated with a mesoscopic device using a sequential deposition process^6^. More recently, a series of breakthroughs were made by the Seok group using mesoscopic devices through solvent^7^, composition^8^ and process engineering^2^. On the other hand, device simplicity and process compatibility would benefit from eliminating the mesoscopic layer and it has been suggested that all devices may ultimately adopt the simple planar architecture^9^. Recently, impressive advances in planar heterojunction PSCs have been realized using organic ETLs^10^. However, the combination of high PCE and long-term stability in these devices remains elusive^11^. Meanwhile, planar PSCs based on compact TiO_2_ are still plagued by lower PCEs and larger current–voltage (JV) hysteresis than the mesoscopic embodiments. It has been suggested that the origin of this performance gap is the much higher electron extraction efficiency from mesostructured TiO_2_ layers^9^. Therefore, improving the electron extraction efficiency from compact TiO_2_ layers could provide a path to simultaneously achieving high efficiency and improved stability by overcoming basic performance limitations in planar heterojunction PSCs.

Although compact TiO_2_ layers are crucial components of planar PSCs, little attention has been paid to understanding and controlling how the characteristics of this material affect device performance. These layers are usually deposited using solution-based processes, such as spin coating and spray pyrolysis, with limited control over their optoelectronic properties. For example, electrical conductivities of compact TiO_2_ films are known to be one order of magnitude lower than those of commonly used organic hole transport layers (HTLs)^12^.

In this work, we consider that the TiO_2_ layer is illuminated during operation of PSCs and show that the photoexcited state properties of compact ETLs play critical roles in defining device performance in terms of efficiency and stability. Both the photoconductivity and photocatalytic activity of TiO_2_ are greatly affected by deep-level trap states associated with native point defects. Here, we have engineered a defective TiO_2_ thin film containing such hole trap states and used it as the ETL for planar heterojunction PSCs. Photoconductive measurements show that the defective TiO_2_ thin film has an internal gain higher than 10^3^ at a small bias of 0.5 V, which is attributed to the minority carrier trapping mechanism in the film. By coupling the highly photoconductive TiO_2_ ETL with a high-quality perovskite light absorber synthesized using a low-pressure vapour annealing process, a highly efficient planar heterojunction PSC with relatively small hysteresis is achieved. The maximum PCEs under reverse scan, forward scan and steady state are 19.0, 17.1 and 17.6%, respectively. Thus, the performance gap between mesoscopic and planar TiO_2_-based devices has been significantly narrowed. Moreover, the stability of the planar heterojunction PSC is greatly improved by using defective TiO_2_ as the ETL. TiO_2_ is known to be an effective ultraviolet photocatalyst for oxidizing organic materials and the hybrid perovskite can be photocatalytically degraded under ultraviolet illumination, which typically leads to poor stability for the PSCs. The incorporation of hole-trap states in the TiO_2_ ETL reduces its activity for photocatalytic oxidation. As a result, the stability of fabricated planar heterojunction PSCs is greatly improved and we show, for the first time, steady-state PCE >15% during continuous operation for 100 h. These results demonstrate that engineering defects in the TiO_2_ ETL is a promising way to improve the efficiency and stability of planar heterojunction PSCs.

## Results

### Photoconductive properties of defective TiO_2_

**Figure 1:**
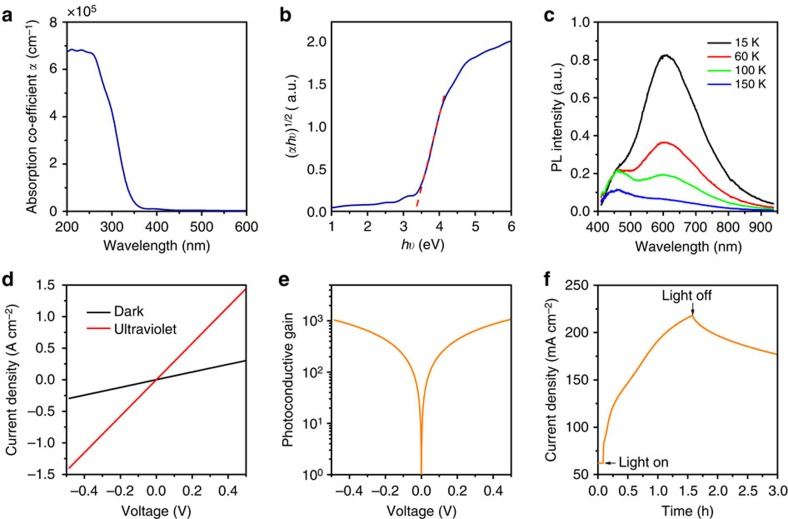
Optical and optoelectronic properties of defective TiO_2_ thin films. (**a**) Absorption coefficient of the TiO_2_ thin film on a Si substrate, determined by spectroscopic ellipsometry. (**b**) Tauc plot for evaluation of indirect optical bandgap, which yields a value of about 3.3 eV. (**c**) Low-temperature PL of TiO_2_ thin film on quartz substrate under 350 nm laser excitation. (**d**) *JV* characteristics of TiO_2_ thin film photoconductor in the dark and under ultraviolet illumination (365 nm, 3.6 mW cm^−2^). (**e**) Photoconductive gain of the TiO_2_ thin film under ultraviolet illumination. (**f**) Time-dependent photoresponse of the TiO_2_ thin-film photoconductor measured at a bias of 0.1 V in N_2_ atmosphere under AM 1.5G illumination.

The compact TiO_2_ thin films used in this work were deposited using electron beam (EB) evaporation with a substrate temperature of 350 °C in an oxygen-deficient environment. We note that this substrate temperature is considerably lower than that required for the formation of mesoscopic TiO_2_ films. X-ray diffraction confirms that the as-deposited TiO_2_ thin films are anatase phase (Supplementary Fig. 1). The thin films are characterized by strong absorption in the ultraviolet region and very low absorption in the visible region, as determined by spectroscopic ellipsometry (Fig. 1a). An indirect bandgap of ∼3.3 eV is extracted from the Tauc plot of the absorption spectrum (Fig. 1b), consistent with that of anatase TiO_2_. The high transmittance in the visible range is important for its application as an ETL, as the perovskite light absorber in the solar cell is illuminated through this layer. Despite low sub-bandgap absorption, temperature-dependent photoluminescence (PL) reveals a broad emission centred at around 600 nm (Fig. 1c). Consistent with prior reports, this defect-related emission from anatase TiO_2_ is very weak at room temperature, indicating that dominant recombination processes are non-radiative^13^. However, this weak radiative recombination indicates the presence of deep-level native defects within the material that are related to oxygen vacancies and/or under-coordinated Ti sites. This finding is consistent with composition analysis by X-ray photoelectron spectroscopy (Supplementary Fig. 2), which reveals that the low-temperature EB-deposited TiO_2_ thin films are slightly (∼2 at%) deficient in oxygen.

**Figure 2:**
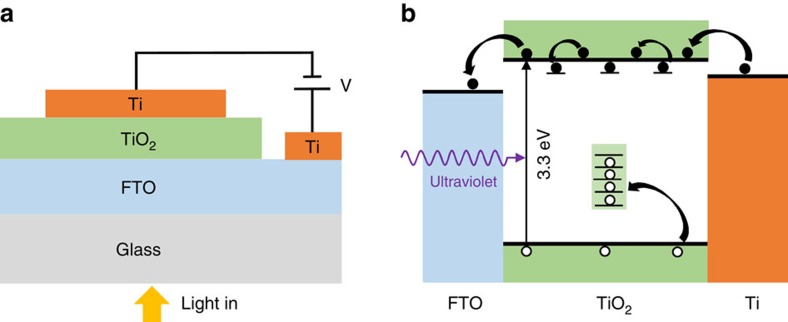
Shematic structure and mechanism of the TiO_2_ photoconductor. (**a**) Schematic structure for characterization of the TiO_2_ thin film as a photoconductor. (**b**) A schematic of the mechanism of defect-meditated photoconductivity in the TiO_2_ thin film.

The presence of deep-level trap states in the TiO_2_ thin film could have a significant impact on its photoconductive properties and, thus, on PSC performance. To explore the role of defects on the photoconductivity of the TiO_2_ thin films, measurements were performed using a photoconductor made by depositing a Ti metal top contact on the TiO_2_ thin film on a fluorine-doped tin oxide (FTO)/glass substrate, as shown in Fig. 2a. For comparison, the conductivity of a conventional solution-processed TiO_2_ film was also measured using the same device architecture (Supplementary Fig. 3). The linear current–voltage (JV) curve measured in the dark indicates Ohmic contacts are formed at both FTO/TiO_2_ and TiO_2_/Ti interfaces (Fig. 1d). The conductivity of the TiO_2_ thin film was greatly increased under ultraviolet illumination. At a bias of 0.5 V, an unexpectedly large photocurrent density of ∼1.1 A cm^−2^ was generated under 365 nm illumination at an irradiance of 3.6 mW cm^−2^. This value greatly exceeds the light-limited photocurrent density that can be generated; even at an external quantum efficiency (EQE) of 100%, the photocurrent density should not exceed 1.1 mA cm^−2^ under these conditions. The observation of an exceptionally high photocurrent density, which is not observed for the case of the conventional solution-processed TiO_2_ film, indicates that there exists a mechanism for internal photoconductive gain in the TiO_2_ thin film. Indeed, as shown in Fig. 1e, a photoconductive gain of more than 10^3^ is obtained at a bias of 0.5 V. Such high photoconductive gain in TiO_2_ has been reported for single crystalline nanorods^14^, but not for polycrystalline thin films. Examination of the temporal evolution of the photoconductivity reveals slow rise and fall times on the order of hours, as shown in Fig. 1f. Such behaviour is characteristic of persistent photoconductivity and, together with the large photoconductive gain, indicates a hole-trapping mechanism in the defective TiO_2_ thin film that results in photo-induced accumulation of excess majority carriers, as shown schematically in Fig. 2b. These electrons contribute to the improved conductivity before eventually recombining with the trapped holes, with long recombination times due to either atomic-scale reorganization following hole trapping at the defect site or spatial segregation of the two carrier types (for instance, trapping of holes at grain boundaries or surfaces).

### Photovoltaic performance of PSCs using defective TiO_2_ as ETL

Defective TiO_2_ thin films exhibiting high photoconductive gain were employed as ETLs for the fabrication of planar heterojunction PSCs. Halide perovskite light-absorbing layers were synthesized using a modified low-pressure vapour annealing process (see Methods section)^15^,^16^. Briefly, mixed lead iodide (PbI_2_) and lead bromide (PbBr_2_) precursor films were annealed in methylammonium iodide (MAI) vapour to produce bromine-doped methylammonium lead iodide perovskites (MAPbI_3-*x*_Br_*x*_). This method yielded high-quality perovskite thin films with large-area uniformity and excellent optical properties (Supplementary Fig. 4). Although most of the bromine in the precursor film was replaced via exchange with iodine in the MAI vapour, a low concentration of bromine was detected in the synthesized perovskite films (Supplementary Fig. 5). The incorporation of a small amount of bromine was found to greatly enhance the stability of the perovskite in ambient atmosphere (Supplementary Fig. 6), consistent with previous findings from solution-processed MAPbI_3-*x*_Br_*x*_ (ref. 7). In addition, it has been demonstrated that the photovoltaic performance of perovskite-based devices can be greatly improved by low-level bromine incorporation^17^. Therefore, MAPbI_3-*x*_Br_*x*_ perovskite films are used for the fabrication of efficient planar heterojunction PSCs in the present work.

**Figure 3:**
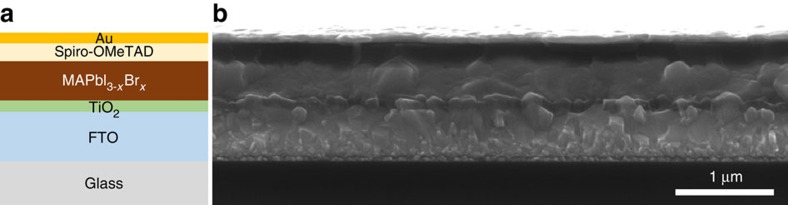
Structure of the planar heterojunction PSC. (**a**) Schematic structure and (**b**) corresponding cross-sectional scanning electron microscopy image of the fabricated planar heterojunction PSC.

**Figure 4:**
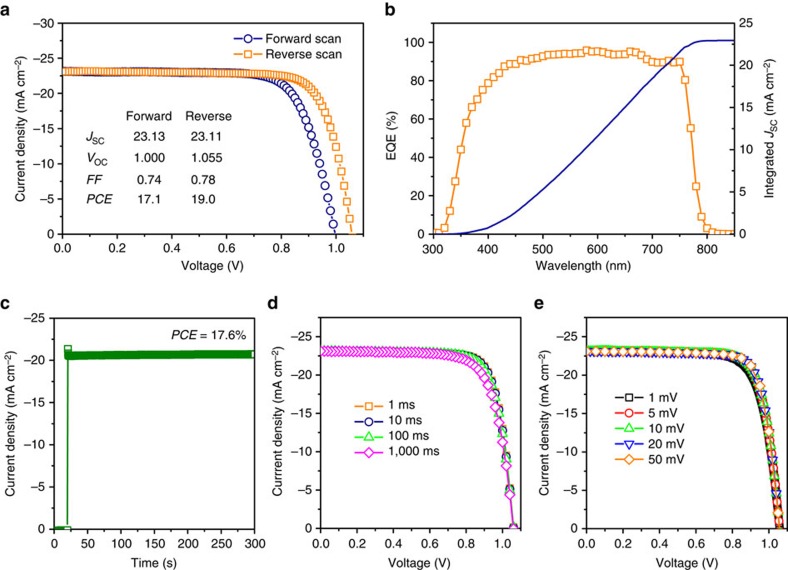
Device performance of the planar heterojunction PSC. (**a**) *JV* curves of one of the highest-performing devices measured in the forward and reverse scan directions at a rate of 10 mV per step under 100 mW cm^−2^ AM 1.5G illumination. (**b**) EQE spectrum of the solar cell measured at the short-circuit condition (orange squares). The integration of the EQE spectrum with the AM 1.5G photon flux is also shown (blue line) and agrees to within 1% of the short circuit current density obtained from *JV* measurements. (**c**) Steady-state measurement of the photocurrent near the maximum power point at 0.85 V. (**d**) *JV* curves of the same device measured with different step delay times and (**e**) voltage step sizes. All the above measurements were carried out after light soaking for ∼20 min.

By coupling highly photoconductive TiO_2_ ETLs with MAPbI_3-*x*_Br_*x*_ perovskites, we demonstrate planar heterojunction PSCs with efficiencies rivaling those of the best mesoscopic devices. The schematic structure and the cross-sectional scanning electron microscopy image of the fabricated planar heterojunction PSCs are shown in Fig. 3a,b. The device architecture consists of an FTO layer on glass substrate as the bottom transparent electrode, an EB-deposited defective TiO_2_ layer (∼100 nm) as the ETL, a MAPbI_3-*x*_Br_*x*_ perovskite layer (∼400 nm) as the light absorber, a spin-coated Spiro-OMeTAD (2,2',7,7'-tetrakis-(*N*,*N*-di-*p*-methoxyphenylamine)9,9'-spirobifluorene) layer (∼200 nm) as the HTL and an EB-deposited gold layer (∼100 nm) as the top electrode. The JV curves in Fig. 4a were obtained after light soaking of one of the highest performing devices. Under reverse scan, the device yielded a short-circuit current density (*J*_SC_) of 23.11 mA cm^−2^, an open-circuit voltage (*V*_OC_) of 1.055 V and a fill factor (FF) of 0.78. A maximum PCE of 19.0% is extracted from the *JV* curve with AM 1.5G illumination at an intensity of 100 mW cm^−2^. Edge effects were found to be small in the PSCs (Supplementary Fig. 7). The EQE spectrum of the planar PSC is shown in Fig. 4b. The data were collected following 20 min of full spectrum light soaking and the photocurrent at each wavelength was measured at steady state after 30 s illumination, to eliminate the influence of transient photocurrents (Supplementary Fig. 8). High EQE values of ∼90% were observed in the visible range, demonstrating the high electron extraction efficiency enabled by the defective TiO_2_ ETL. Integration of the EQE spectrum with the AM 1.5G (ASTM G173-03 reference spectrum) photon flux yields a photocurrent density of 22.93 mA cm^−2^, which agrees to within 1% of the values obtained from the *JV* curves presented in Fig. 4a.

Although hysteresis is observed for this planar device, it is much smaller than is found in other PSCs based on planar TiO_2_ (refs 7, 12, 18). Under forward scan, although the *J*_SC_ was almost unchanged, slightly reduced *V*_OC_ and FF were observed, leading to a lower PCE of 17.1% at the maximum power point. To determine the steady-state PCE, the device was held at an external bias close to the maximum power point and the current was recorded. As shown in Fig. 4c, a stable photocurrent density of 20.7 mA cm^−2^ was observed at a bias of 0.85 V, indicating that the steady-state PCE of the device is at least 17.6%. It was confirmed that the steady-state PCE lies between the values extracted from JV curves measured in the reverse and forward scan directions (Supplementary Fig. 9). The stable steady-state photocurrent observed here is also improved relative to the steadily decaying photocurrent observed in previously reported planar devices^18^. In that work, the photocurrent decay under steady-state operation was ascribed to a reduction of electron extraction efficiency of the planar TiO_2_ layer^18^. The stable steady-state photocurrent in our case suggests that the electron extraction efficiency of the TiO_2_ ETL remained high during operation. The high electron extraction efficiency of the TiO_2_ ETL is also supported by the fact that the hysteresis is relatively small in our devices. As shown in Fig. 4d,e, the JV curves do not show a strong dependence on the step size or the delay time during testing. Although the reduced hysteresis in mesoscopic PSCs remains a topic of investigation, some reports have ascribed it to the enhanced electron extraction efficiency of the TiO_2_ layer^9^. By increasing the conductivity of the planar TiO_2_ layer, the electron extraction efficiency is also improved, thereby contributing to a reduced hysteresis in the fabricated planar PSCs. The enhanced electron extraction efficiency of the EB-deposited TiO_2_ thin film compared with the conventional solution-processed TiO_2_ thin film was further confirmed via PL quenching measurements (Supplementary Fig. 10). The performance of a planar PSC fabricated using solution-processed TiO_2_ as the ETL is given in Supplementary Fig. 11 for reference.

Table 1 summarizes the photovoltaic performance from 50 planar heterojunction PSCs fabricated using defective TiO_2_ as the ETL. Corresponding box charts of the data are given in the Supplementary Fig. 12. In the reverse scan direction, the average values of *J*_SC_, *V*_OC_ and FF were 22.5 mA cm^−2^, 1.05 V and 0.74, respectively, yielding an average PCE of 17.5%. In the forward scan direction, average values of *V*_OC_ and FF dropped to 1.00 V and 0.70, respectively, leading to a lower average PCE of 15.8%. Hysteresis was evaluated by comparing the PCE values obtained under reverse and forward scan [(PCE_reverse_−PCE_forward_)/PCE_reverse_], which yielded values <10%. Importantly, standard deviation of all photovoltaic performance metrics were small, exhibiting values of <6%. These results indicate that planar heterojunction PSCs fabricated using the defective TiO_2_ provide high efficiency, low hysteresis and good reproducibility.

### Improved electron extraction efficiency by defective TiO_2_

The large photoconductivity of the defective TiO_2_ ETL is essential for achieving high efficiency with low hysteresis. For comparison, planar heterojunction PSCs were also fabricated using TiO_2_ thin films annealed at 500 °C in air as the ETL. The photoconductivity of the EB-deposited TiO_2_ thin films decreased drastically after annealing (Supplementary Fig. 13). This finding is consistent with increased electron-hole recombination rates in the annealed TiO_2_ due to reduced concentrations of beneficial long-lived hole-trap states associated with photoconductive gain and persistent photoconductivity. Consequently, the photovoltaic performance of the planar PSCs fabricated using post-annealed TiO_2_ thin films as ETL was lowered (Supplementary Fig. 14). Compared with PSCs fabricated with as-deposited TiO_2_, PSCs fabricated with post-annealed TiO_2_ yielded lower FF (Supplementary Table 1), which is consistent with the increased series resistance due to the decreased photoconductivity of the TiO_2_ ETL. The larger hysteresis that is observed following annealing is also in agreement with the reduced electron extraction efficiency due to the decreased photoconductivity.

**Figure 5:**
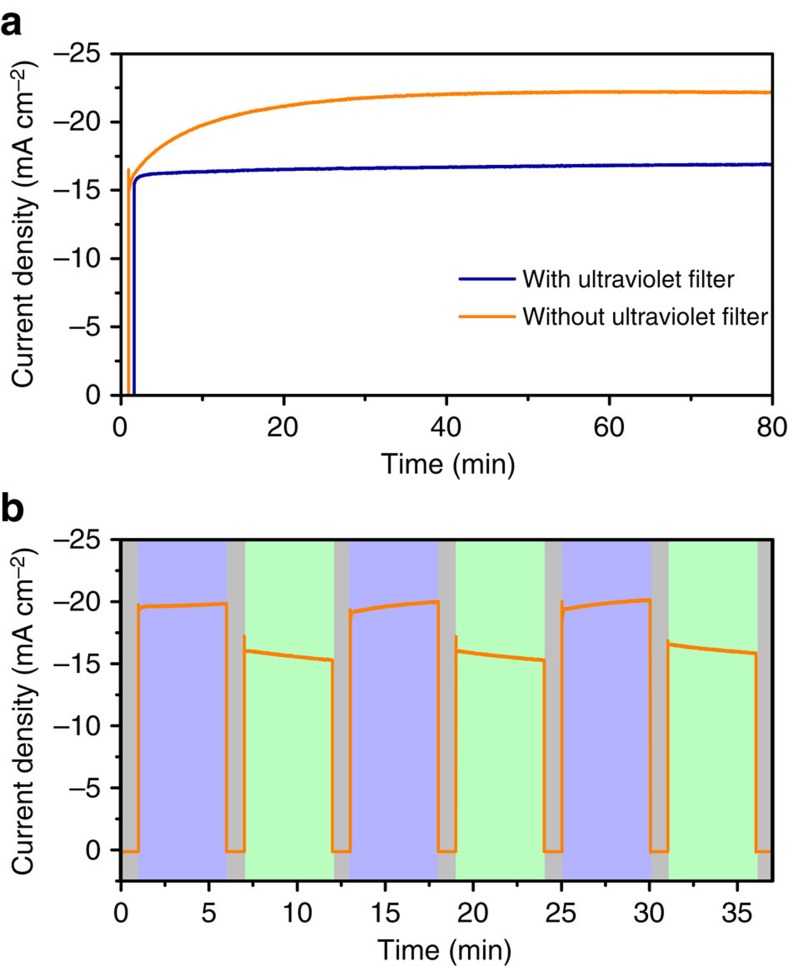
Effect of the hole traps in the defective TiO_2_ on the photocurrent of planar heterojunction PSC. (**a**) Evolution of the photocurrent from a complete PSC under full AM 1.5G spectrum (orange line) and visible light (blue line) illumination. The curves were measured at a bias of 0.8 V and a 425 nm-long pass filter was used to block the ultraviolet light. The devices were not subjected to any illumination before the test. (**b**) Charge trapping and de-trapping process revealed in complete PSCs by repeatedly applying and removing the ultraviolet filter. The curve was measured at a bias of 0.85 V after light soaking for about 30 min. Grey colour: in the dark, purple colour: under full AM 1.5G illumination, green colour: under visible illumination.

As the conductivity of the TiO_2_ is mainly affected by ultraviolet illumination (Supplementary Fig. 15), the effect of hole trapping in the TiO_2_ ETL on the photovoltaic properties of the planar PSCs was investigated by measurement with and without an ultraviolet cutoff (425 nm-long pass) filter. As discussed above and shown in Fig. 1f, under full spectrum illumination, the photocurrent from the TiO_2_ thin film photoconductor increased and decreased gradually when the light was switched on and off, respectively. Likewise, the photocurrent of the complete PSC increased gradually on illumination with full-spectrum AM 1.5G light, as shown in Fig. 5a. In contrast, no obvious light-soaking process was observed when ultraviolet light was blocked from the illumination (Fig. 5a). Therefore, the effect of light soaking on increasing photocurrent in complete PSCs under full spectrum illumination is attributed to the gradually increased conductivity of the TiO_2_ ETL in the presence of ultraviolet illumination, which is enabled by incorporation of long-lived deep-level hole trap states. This assignment is also supported by the absence of a significant light-soaking process under AM 1.5G illumination in planar PSCs fabricated using post-annealed TiO_2_ thin films as the ETL (Supplementary Fig. 16).

The effect of hole trapping and de-trapping on the photocurrent of PSCs was further revealed by repeatedly applying and removing the ultraviolet filter during measurement. As shown in Fig. 5b, the photocurrent of the PSC gradually increased with ultraviolet illumination due to hole trapping and accumulation of unpaired electrons in the TiO_2_. When the ultraviolet filter was applied, the photocurrent gradually decreased due to recombination of the accumulated electrons with trapped holes. The photocurrent rise and decay with and without ultraviolet illumination can be repeated, because the hole trapping and de-trapping is reversible. These results provide additional support for the finding that the high efficiency achieved in the fabricated planar PSCs is related to the hole trapping in the defective TiO_2_ thin film.

### Improved PSC stability with defective TiO_2_ ETLs

**Figure 6:**
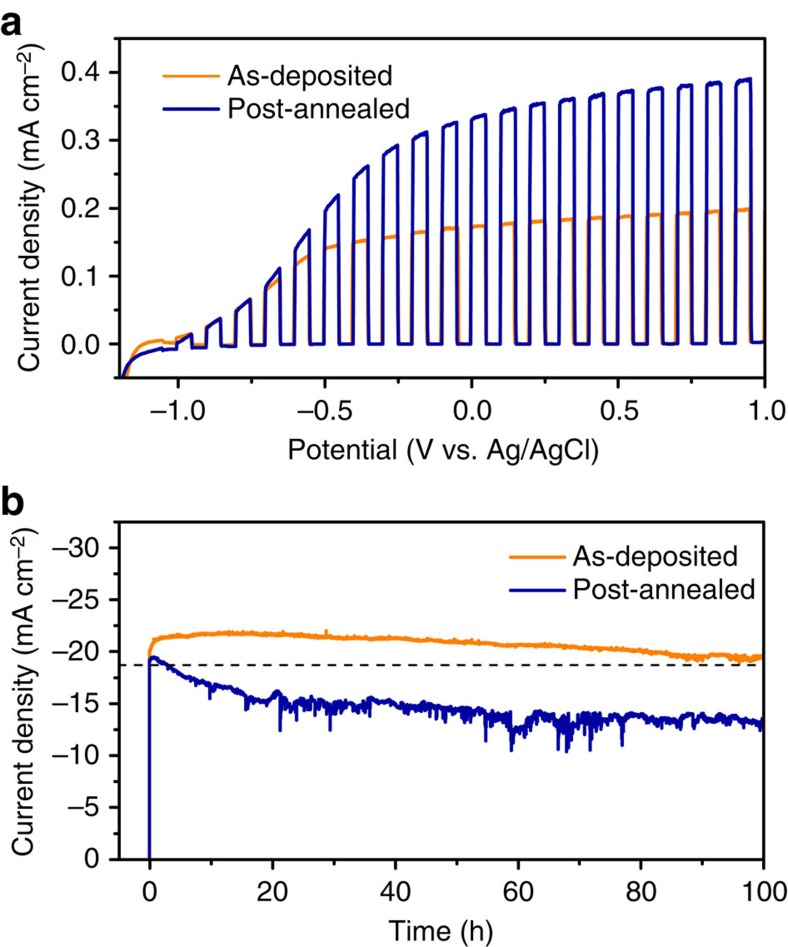
Effect of the hole traps in the defective TiO_2_ on the long-term stability of the planar heterojunction PSC. (**a**) Photoelectrochemical oxidation of methylamine in water using as-deposited (orange line) and post-annealed (blue line) TiO_2_ on FTO glass as photoanodes. The *JV* curves were measured under chopped simulated sunlight (AM 1.5G) and anodic scan at a rate of 10 mV s^−1^. (**b**) Long-term stability of the planar heterojunction perovskite solar cell fabricated using as-deposited (orange line) and post-annealed (blue line) TiO_2_ thin film as the ETL. The curves were measured at a bias of 0.8 V under AM 1.5G in N_2_ atmosphere. The dashed line indicates a PCE of 15%.

A significant concern associated with using TiO_2_ as ETL is that the organic/inorganic hybrid perovskite could be photocatalytically degraded by TiO_2_ on photoexcitation with the ultraviolet portion of the solar spectrum, leading to poor stability for TiO_2_-based PSCs^19^. Several methods have been proposed to overcome this instability, including replacing TiO_2_ with ultraviolet inactive materials such as C_60_ and alumina^20^,^21^, inserting a buffer layer between TiO_2_ and perovskite^22^, and using ultraviolet filters^23^. However, these methods lead to lower device efficiencies due to reduced electron extraction efficiencies and/or parasitic light absorption. As photogenerated holes in the TiO_2_ are required to facilitate oxidation of organic materials^24^, incorporation of deep-level hole traps into defective TiO_2_ provides opportunity to suppress photocatalytic activity and increase the stability of the TiO_2_/perovskite interface. We tested the photocatalytic activity of defective TiO_2_ thin films by photoelectrochemical oxidation of methylamine (CH_3_NH_2_) in aqueous solution. As shown in Fig. 6a, the photocurrent of the defective as-deposited TiO_2_ thin film was much lower than that of the post-annealed sample, which possessed fewer long-lived hole trap states. This decrease in the photocatalytic activity of the defective TiO_2_ thin film has important implications for stability of functional photovoltaic devices. Indeed, the planar PSC employing as-deposited TiO_2_ as the ETL shows dramatically enhanced long-term stability. As shown in Fig. 6b, the photocurrent decayed by only about 10% from its maximum value after continuous operation near the maximum power point for 100 h. In contrast, the photocurrent of the PSC fabricated using post-annealed TiO_2_ decayed by >30% after 100 h. In addition, the planar PSC fabricated using defective TiO_2_ yielded a maximum PCE of 17.6% and PCEs over 15.4% for the entire 100 h testing period. To the best of our knowledge, this combination of high efficiency (over 15%) with long-term stability has not been demonstrated with either planar or mesoscopic PSCs. The results suggest that employing defective TiO_2_ with hole traps as the ETL is an effective way to enhance the stability of the planar PSCs while maintaining, and even promoting, the high efficiency of the device.

## Discussion

We find that incorporation of deep-level hole traps in EB-deposited TiO_2_ thin films leads to an unexpectedly large photoconductive gain under ultraviolet illumination. The highly photoconductive TiO_2_ thin film was employed as the ETL for a planar heterojunction PSC. The large photoconductivity of this layer improves electron extraction efficiency in planar PSC devices, increases the PCE and reduces JV hysteresis. In addition, the hole traps in the TiO_2_ thin film reduce its ultraviolet photocatalytic activity for degradation of the hybrid perovskite. As a consequence, the long-term stability of planar PSCs is significantly improved and yields PCEs >15.4% for at least 100 h, for the first time. Furthermore, the beneficial effects of defect incorporation are achieved with a maximum temperature of 350 °C, which is significantly lower than is normally required for processing of mesoscopic TiO_2_ ETLs. Our results demonstrate an effective way to not only enhance the efficiency but also improve the stability of planar heterojunction PSCs by engineering defects in TiO_2_ ETLs.

## Methods

### TiO_2_ thin film deposition

Compact TiO_2_ thin films (100 nm) were deposited on fluorine-doped tin oxide-coated glass (FTO/glass), quartz or silicon substrates by EB evaporation (Angstrom Engineering, NEXDEP). Before deposition, the chamber was evacuated to a base pressure of ∼2 × 10^−6^ Torr and the substrate was heated to 350 °C. TiO_2_ pieces (Kurt J. Lesker, 99.9% purity) were used as the source for evaporation. The deposition was conducted under an acceleration voltage of 7 kV and the current was adjusted to reach a deposition rate of about 0.5 Å s^−1^. Following deposition, the substrates were allowed to cool overnight in vacuum. Post-annealing of the TiO_2_ thin film was carried out in air at 500 °C for 2 h using a tube furnace. Solution-processed TiO_2_ compact layers were deposited by spin coating a mildly acidic solution of titanium isopropoxide in ethanol (350 μl in 5 ml ethanol with 0.013 M HCl) at 2,000 r.p.m. and annealing in air at 500 °C for 30 min.

### Solar cell fabrication

Substrates for solar cell fabrication consisted of compact TiO_2_ ETLs, deposited on FTO/glass, as described above. A mixture of 0.8 M PbI_2_ (Alfa Aesar, 99.9985%) and 0.2 M PbBr_2_ (Sigma-Aldrich, 99.999%) was dissolved in *N*,*N*-dimethylformamide (Sigma-Aldrich, 99.9%) and filtered with a 0.2 μm syringe filter. Mixed PbI_2_/PbBr_2_ films were deposited by spin coating in air at 1,500 r.p.m. for 3 min and dried on a hot plate at 110 °C for 15 min. Samples were then transferred to test tubes charged with 0.1 g of MAI. The tube was evacuated with a rotary pump and heated with a silicone oil bath. The mixed PbI_2_/PbBr_2_ film was annealed in MAI vapour at 120 °C for 2 h under a pressure of ∼0.4 Torr, to form MAPbI_3-*x*_Br_*x*_. The perovskite films were then washed with isopropyl alcohol (Sigma-Aldrich, 99.5%) and the HTL was applied immediately afterwards. The precursor solution for the HTL was prepared by dissolving 80 mg spiro-OMeTAD (Lumtec, 99.5%), 28.5 μl 4-*tert*-butylpyridine (Sigma-Aldrich, 96%) and 17.5 μl lithium-bis(trifluoromethanesulfonyl)imide (Sigma-Aldrich, 99.95%) solution (520 mg lithium-bis(trifluoromethanesulfonyl)imide in 1 ml acetonitrile) in 1 ml chlorobenzene (Sigma-Aldrich, 99.8%). The HTL was deposited by spin coating at 3,000 r.p.m. for 30 s in air. Samples were then stored in a desiccator overnight. A 100-nm-thick Au layer was then deposited on top of the HTL through a metal shadow mask by EB evaporation at a base pressure of about 2 × 10^−6^ Torr and a deposition rate of about 2 Å s^−1^. The active area for all solar cells was 0.16 cm^2^.

### Characterization

X-ray diffraction spectra were measured with a Rigaku SmartLab X-ray diffractometer using Cu K_α_ radiation at 40 kV and 40 mA. Spectroscopic ellipsometry data were collected on a J.A. Woollam Co. M-2000 ellipsometer with extended NIR range. Data fitting was performed with CompleteEASE software to extract the absorption spectrum. Scanning electron microscopy images of the samples were acquired using a FEI QUANTA FEG 250. Ultraviolet–vis spectra of the perovskite films were measured with a Shimadzu SolidSpec-3700 spectrometer. PL spectra of TiO_2_ thin films were recorded using an Andor spectrometer equipped with 600 lines per mm grating with 500 nm blaze under 350 nm excitation using a Coherent OPerA Solo optical parametric amplifier pumped with a Coherent Libra amplified Ti:S system. Photoconductivity data from TiO_2_ thin films were collected using a potentiostat (BioLogic SP-200) and an ultraviolet lamp (365 nm, 3.6 mW cm^−2^) in N_2_ atmosphere. *JV* curves and steady-state photocurrent data of the solar cells were measured in air using a solar simulator (Newport, 91192) equipped with a 150 W Xe lamp and an AM 1.5G filter as light source and a Keithley 2400 source meter. The light intensity was calibrated with an NREL-calibrated Si solar cell with a KG-5 filter to 1 sun (100 mW cm^−2^). The EQE spectrum was measured at zero bias under continuous illumination by monochromatic light obtained using a 300 W Xenon lamp and a monochromator. The incident beam was focused within the active area of the device. Long-term stability of solar cells was measured under 100 mW cm^−2^ simulated sunlight (Solar Light 16S-300-005) in N_2_ atmosphere. The spectrum of the solar simulator used for long-term stability testing and the light-induced temperature increase during stability testing are shown in Supplementary Fig. 17. Photoelectrochemical properties of TiO_2_ thin films were measured with a three-electrode configuration using a Ag/AgCl reference electrode and a Pt wire counter electrode. An aqueous solution of 1 wt.% methylamine was used as the electrolyte. JV curves were measured under chopped simulated sunlight (Solar Light 16S-300-005) and anodic scan at a rate of 10 mV s^−1^.

### Data availability

The authors declare that all data that support the findings of this study are available within an associated file in the Supplementary Data of this article.

## Additional information

**How to cite this article**: Li, Y. *et al.* Defective TiO_2_ with high photoconductive gain for efficient and stable planar heterojunction perovskite solar cells. *Nat. Commun.* 7:12446 doi: 10.1038/ncomms12446 (2016).

## Supplementary Material

Supplementary Information Supplementary Figures 1-17 and Supplementary Table 1

## Figures and Tables

**Table 1:** Photovoltaic performance of the planar heterojunction PSCs.

Scan direction	**_SC_ (mA cm^−2^)	*V*_OC_ (V)	FF	PCE (%)
Reverse	22.5±0.6	1.05±0.02	0.74±0.02	17.5±1.0
Forward	22.5±0.6	1.00±0.02	0.70±0.03	15.8±0.9

Statistics are given for 50 devices with mean values and standard deviations for the solar cell parameters.
