# Supplementary material for: Defective TiO2 with high photoconductive gain for efficient and stable planar heterojunction perovskite solar cells
Source: Nat Commun. 2016 Aug 18;7:12446. doi: 10.1038/ncomms12446 (PMC4992141; doi:10.1038/ncomms12446)
Supplement: Supplementary Information — Supplementary Figures 1-17 and Supplementary Table 1 [file ncomms12446-s1.pdf]

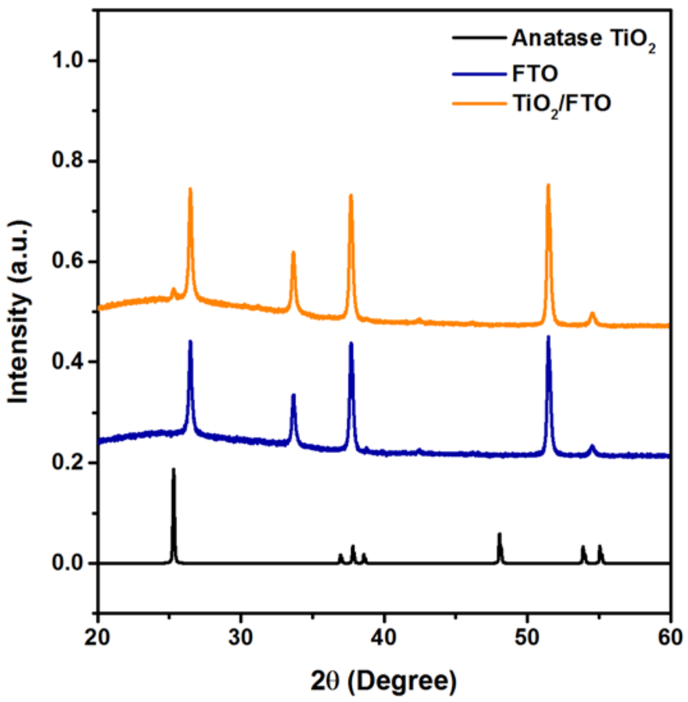

Supplementary Figure 1 | XRD pattern of a defective TiO_2 thin film deposited on an FTO/glass substrate, along with an XRD pattern of bare FTO/glass and a reference pattern of anatase TiO_2 (JSPDS No.: 21-1272).

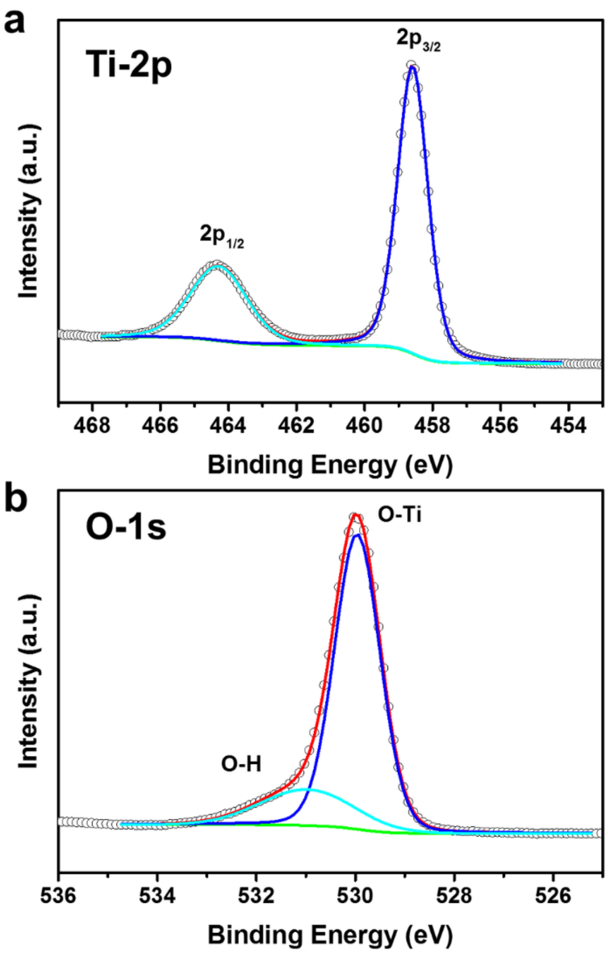

Supplementary Figure 2 | XPS spectra of TiO_2 thin film deposited by EB evaporation. (**a**) Ti 2p peaks and (**b**) O 1s peak. The atomic ratio of Ti:O was calculated to be 1.96:1 using the integrated peak areas and sensitivity factors of 1.2 for the Ti $2p_{3/2}$ peak and 0.66 for the O 1s peak.

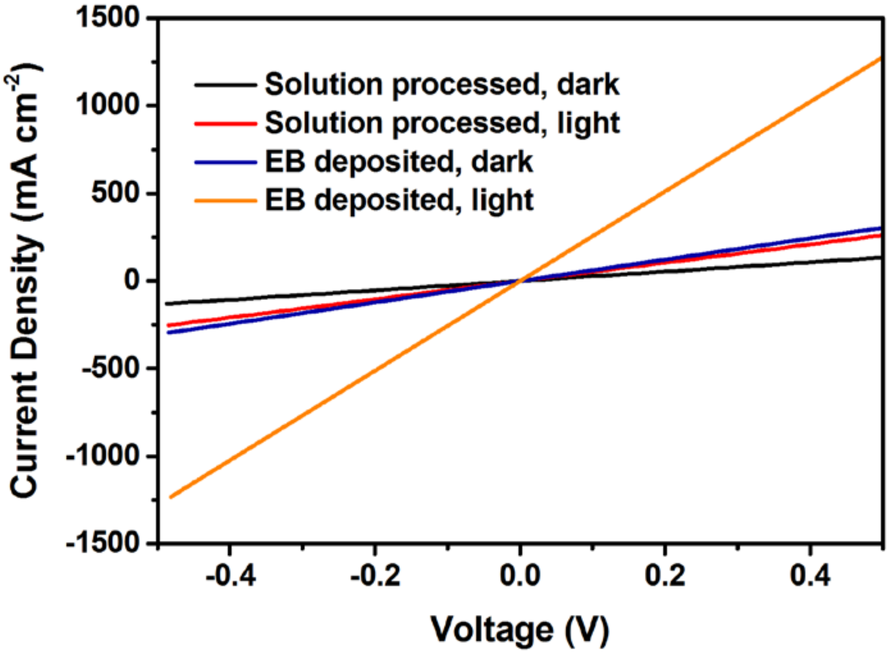

Supplementary Figure 3 | Conductivity of conventional solution processed TiO_2 thin film and electron beam (EB) deposited TiO_2 thin film in the dark and under simulated sunlight (AM 1.5G). The EB deposited TiO_2 thin film showed much higher conductivity, both in dark and under illumination, than the solution processed TiO_2 thin film.

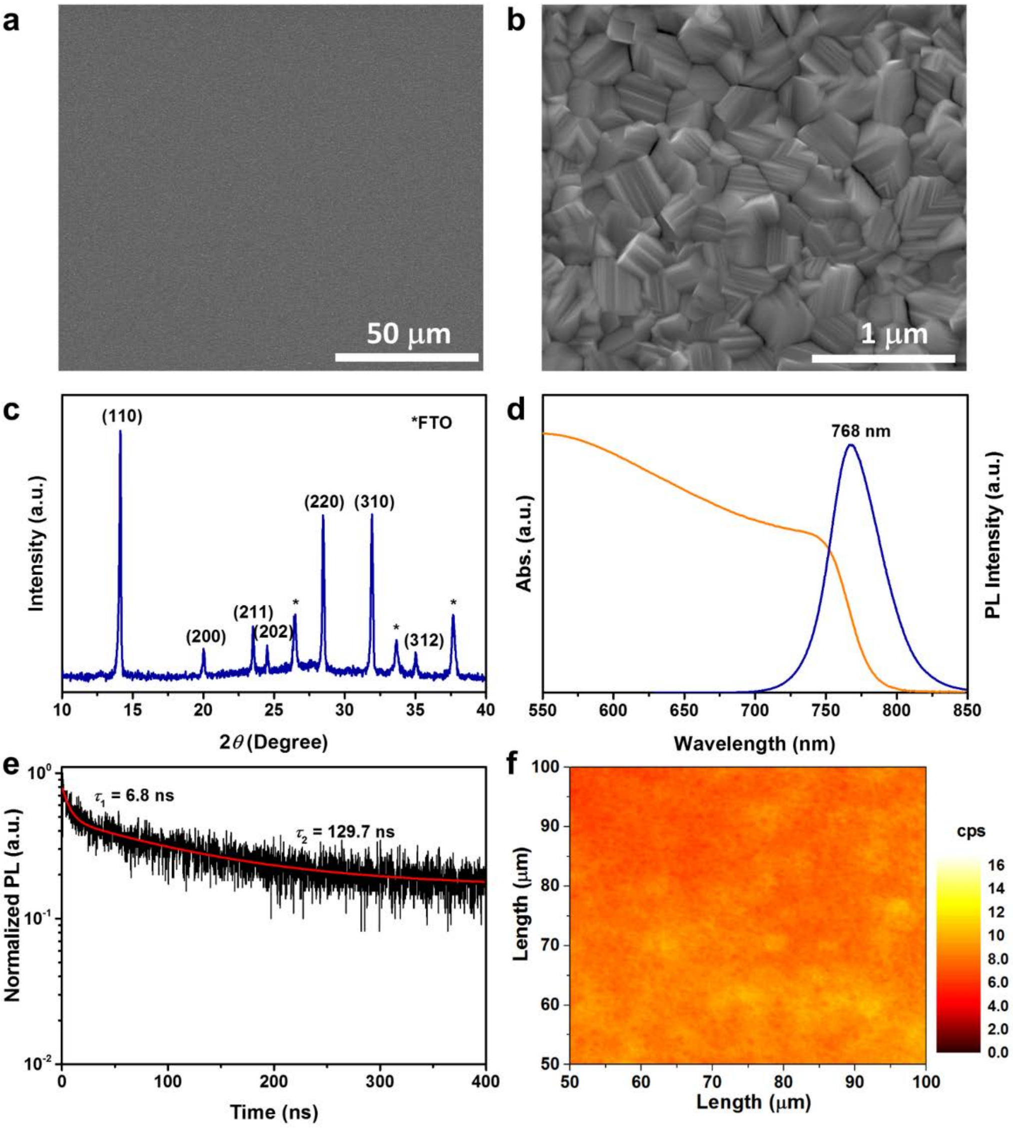

Supplementary Figure 4 | Structural and optical properties of the $CH_3NH_3PbI_{3-x}Br_x$ perovskite thin film synthesized by low-pressure vapor annealing process. (**a**) Low-magnification SEM image of the perovskite thin film showing large-scale uniformity without pinhole defects. (**b**) High-magnification SEM image of the perovskite thin film revealing highly faceted grains with an average size of ~400 nm. (**c**) XRD pattern of the perovskite thin film grown on TiO_2/FTO substrate. (**d**) Absorption spectrum (orange line) and PL spectrum (blue line) of the $CH_3NH_3PbI_{3-x}Br_x$ perovskite. (**e**) Time-resolved PL of the $CH_3NH_3PbI_{3-x}Br_x$ perovskite thin film revealing a bi-exponential decay with a fast component of 6.8 ns and a slow component of 129.7 ns. The curve was measured at 0.02 μJ cm^{-2} laser fluence. (**f**) PL imaging showing large-scale uniformity of the $CH_3NH_3PbI_{3-x}Br_x$ perovskite thin film. The sample gave a mean emission intensity of 8.1 cps with a standard derivative of 0.8 cps.

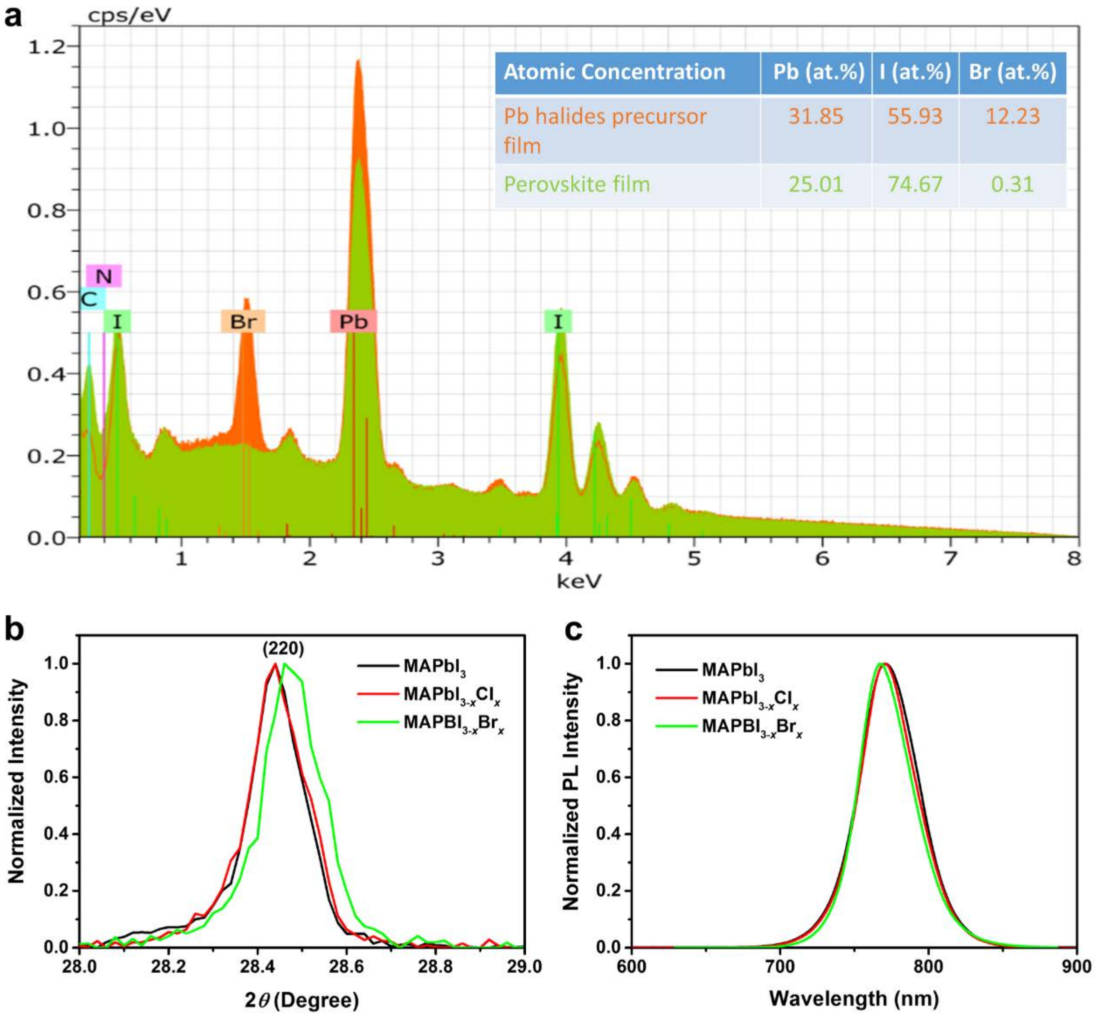

Supplementary Figure 5 | Evidence for the presence of Br in the synthesized perovskite thin film. (**a**) EDX spectrum of the $CH_3NH_3PbI_{3\text{-}x}Br_x$ perovskite thin film (green). EDX spectrum of the mixed lead halide precursor film (orange) is also shown for comparison. The inserted table summarizes the atomic concentration of the Pb, I, and Br in these films. (**b**) XRD spectra of the (220) peak of the perovskites showing a shift to higher 2θ values due to the incorporation of Br. (**c**) PL spectra of the halide perovskite films, revealing a blue shift of the emission peak due to the incorporation of Br. Data from $CH_3NH_3PbI_{3\text{-}x}Cl_x$ films is shown for comparison, since such layers are most commonly investigated for high performance PSCs.

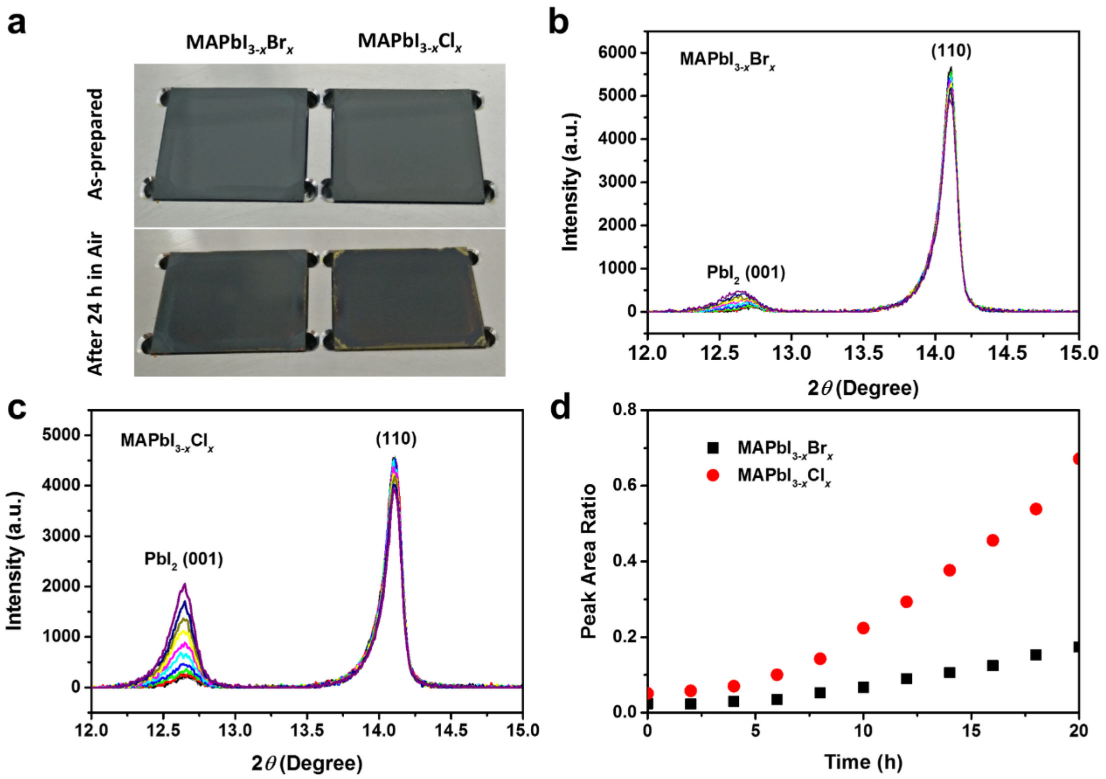

Supplementary Figure 6 | Stability of $CH_3NH_3PbI_{3\text{-}x}Br_x$ and $CH_3NH_3PbI_{3\text{-}x}Cl_x$ perovskite thin films in air. (**a**) Photographs of the as-prepared perovskite thin films before and after storage in air (relative humidity about 50%) for 24 h. A higher degree of degradation was observed in the more commonly investigated $CH_3NH_3PbI_{3\text{-}x}Cl_x$ perovskite thin film compared to the $CH_3NH_3PbI_{3\text{-x}}Br_x$ thin film used here. (**b**) XRD spectra of the $CH_3NH_3PbI_{3\text{-}x}Br_x$ thin film recorded every 2 h. (**c**) XRD spectra of the $CH_3NH_3PbI_{3\text{-}x}Cl_x$ thin film recorded every 2 h. (**d**) Evolution of areal ratios of the PbI_2 (001) peak to the perovskite (110) peak with air exposure time for $CH_3NH_3PbI_{3\text{-}x}Br_x$ and $CH_3NH_3PbI_{3\text{-}x}Cl_x$ thin films.

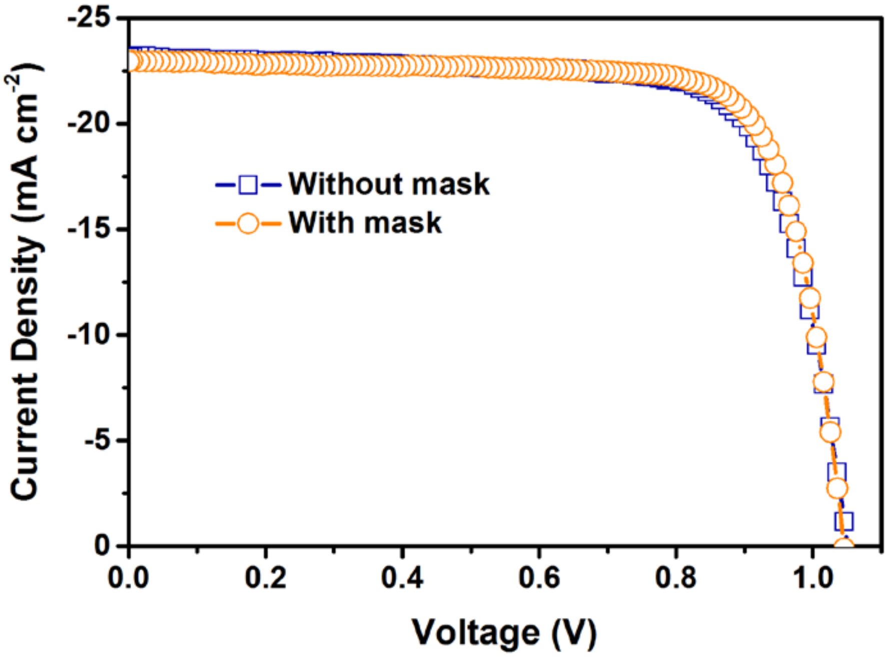

Supplementary Figure 7 | *JV* curves of a PSC measured with and without a physical mask. The active area of the device without a mask was calculated by measuring the overlapped area between the FTO and the Au electrode, which was 0.16 cm^2. The masked area within the active area was 0.106 cm^2. The short circuit current obtained with the mask (22.95 mA cm^{-2}) is very close to the value obtained without the mask (23.15 mA cm^{-2}). The fill factor was observed to increase when illumination was performed through a mask, resulting in an increase of the *PCE* from 18.3% to 18.6%. These results demonstrate that edge effects are small in these devices and do not lead to overestimation of *PCE* values.

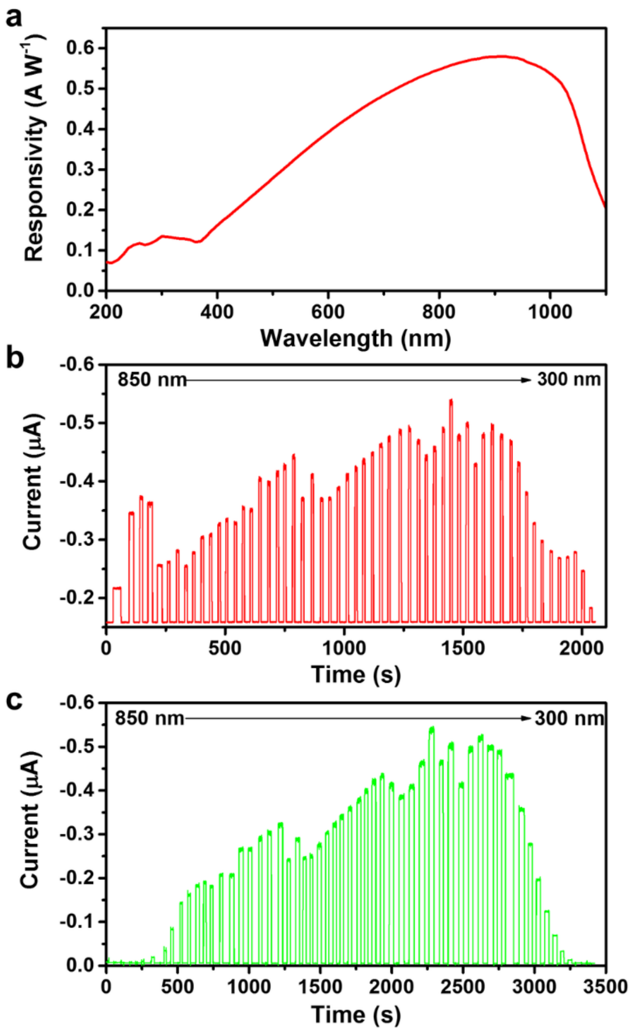

Supplementary Figure 8 | Measurement of *EQE* spectrum for the perovskite solar cell. (**a**) Responsivity spectrum of the reference Si diode. (**b**) Photocurrent of the reference Si diode. (**c**) Photocurrent of the perovskite solar cell.

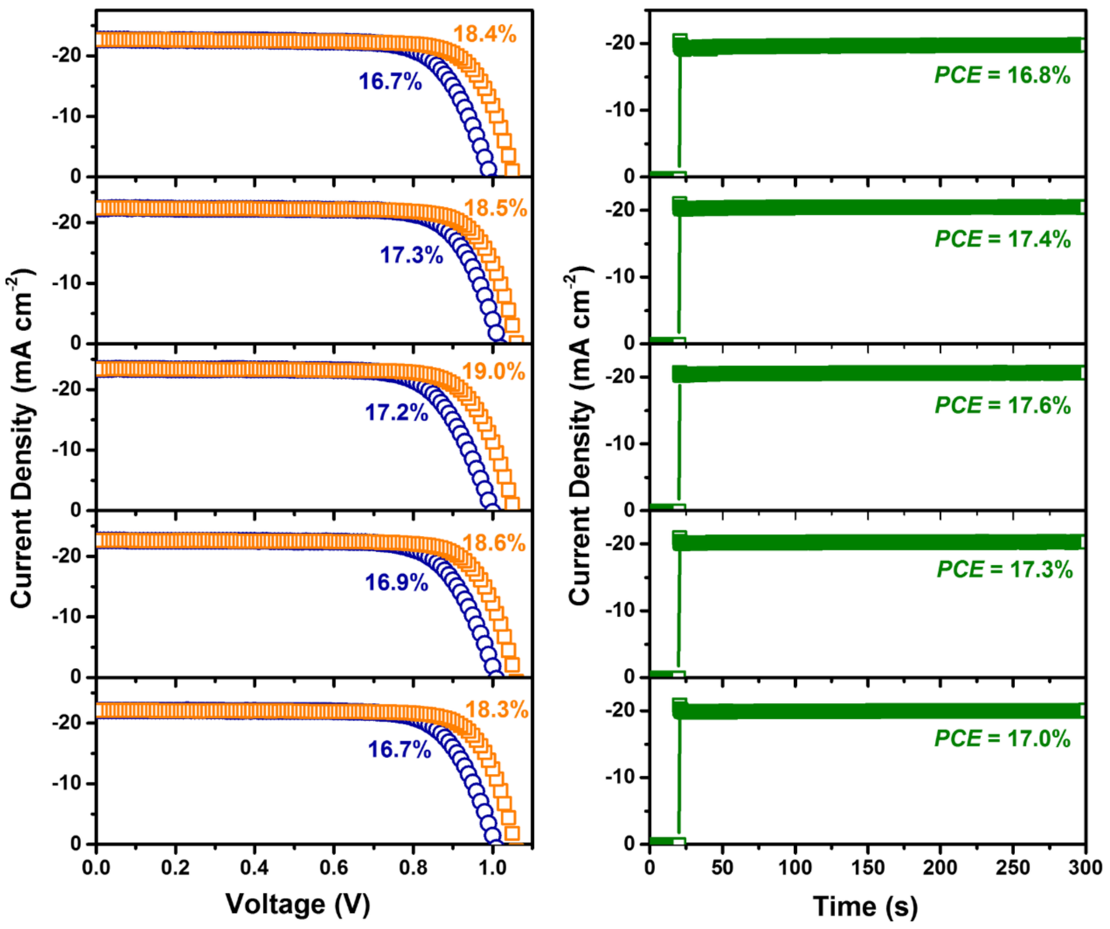

Supplementary Figure 9 | *JV* curves and corresponding steady-state photocurrent near the maximum power point (bias = 0.85 V) for 5 individual devices. The results confirm that small hysteresis and stable steady-state photocurrent are characteristics of the planar heterojunction PSCs fabricated using defective TiO_2 ETL and $CH_3NH_3PbI_{3-x}Br_x$ perovskite film deposited by the low-pressure vapor annealing process. The results also demonstrate that the steady-state *PCE* lies between the values obtained from JV curves acquired under reverse scan and forward scan.

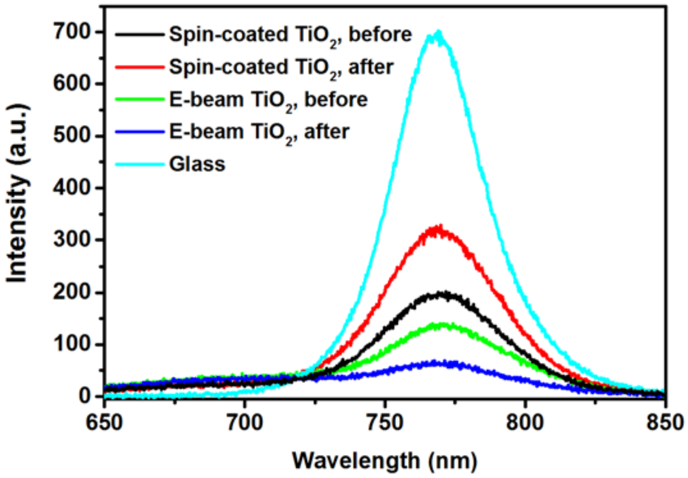

Supplementary Figure 10 | PL spectra of perovskite film deposited on glass, spin-coated TiO_2/FTO glass, and EB deposited TiO_2/FTO glass before and after light soaking under simulated sunlight (AM 1.5G) for 20 min. The lower PL intensity of the perovskite on EB deposited TiO_2 than on spin-coated TiO_2 demonstrates that the EB deposited TiO_2 has better electron extraction efficiency than spin-coated TiO_2 both before and after light soaking. More importantly, it was found that the PL was further quenched after light soaking for the EB deposited sample, demonstrating that the electron extraction efficiency of the EB deposited TiO_2 was improved by light soaking. In contrast, the electron extraction efficiency of the spin-coated TiO_2 was lowered by light soaking, probably due to the degradation of the perovskite at the perovskite/TiO_2 interface.

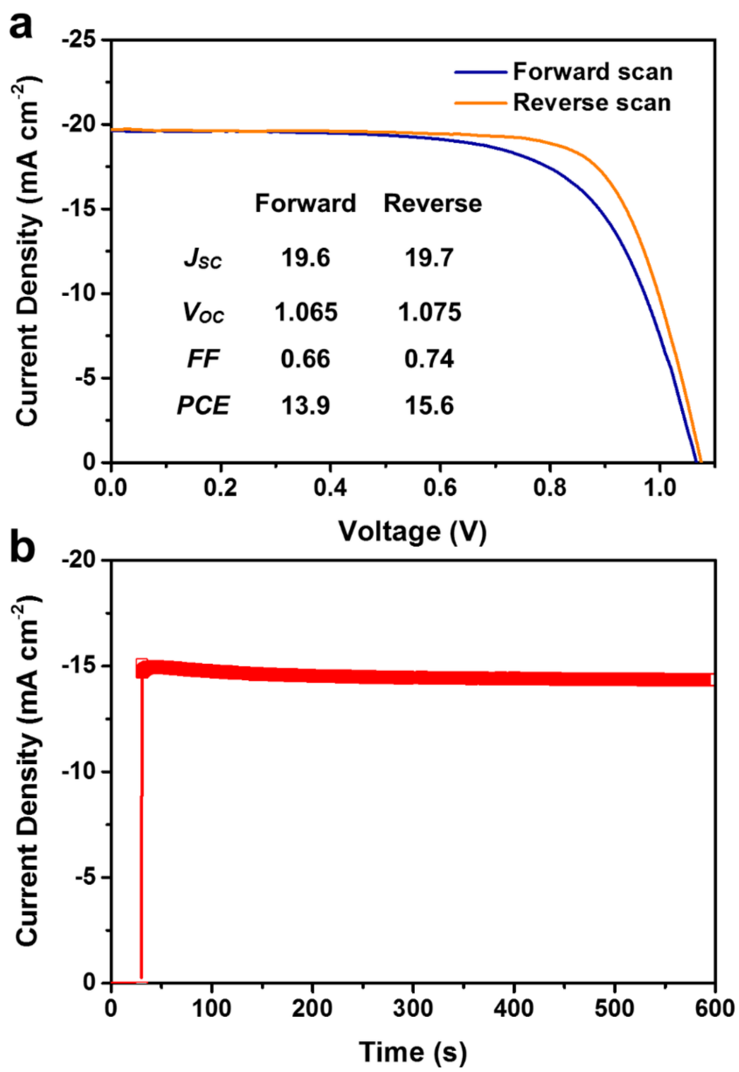

Supplementary Figure 11 | Photovoltaic performance of PSC fabricated using spin-coated TiO_2 thin film as the ETL. (**a**) *JV* curves measured in the forward and reverse scan directions at a rate of 10 mV per step under 100 mW cm^{-2} AM 1.5G illumination. (**b**) Steady state measurement of the photocurrent near the maximum power point at 0.9 V.

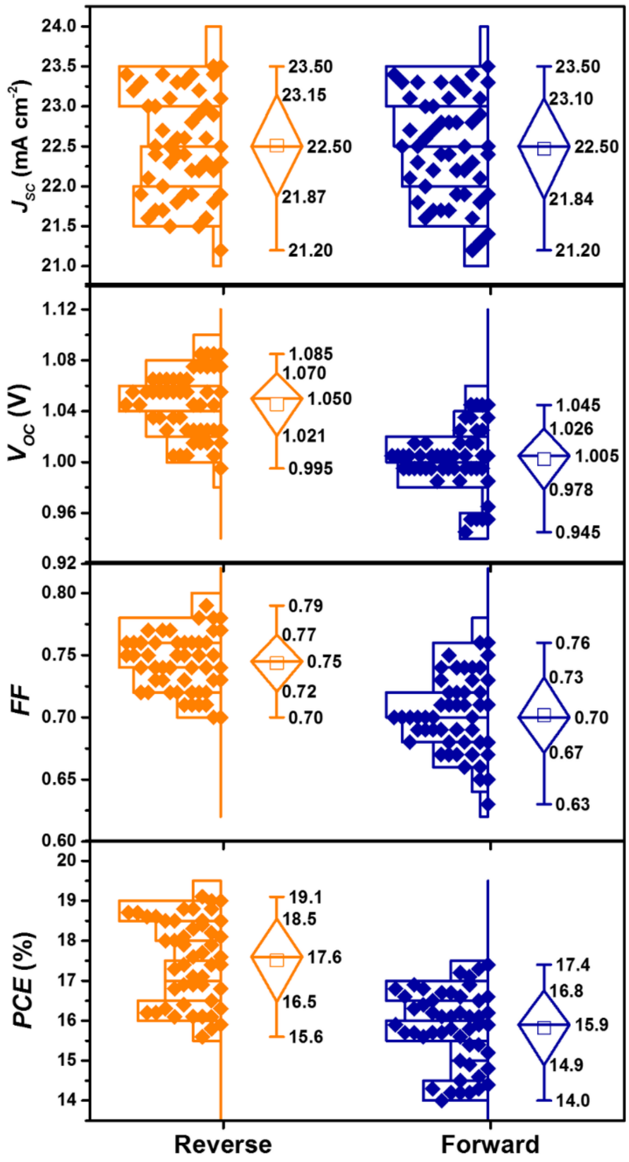

Supplementary Figure 12 | Box charts of J_{SC}, V_{OC}, FF, and PCE obtained under reverse and forward scan for 50 individual PSCs. The values to the right of the boxes indicate (from top to bottom): the maximum, +1 standard deviation, the 50% percentile, -1 standard deviation, and the minimum. The open squares inside of the boxes indicate the mean values. The bars and dots to the left the boxes show the statistics of the raw data.

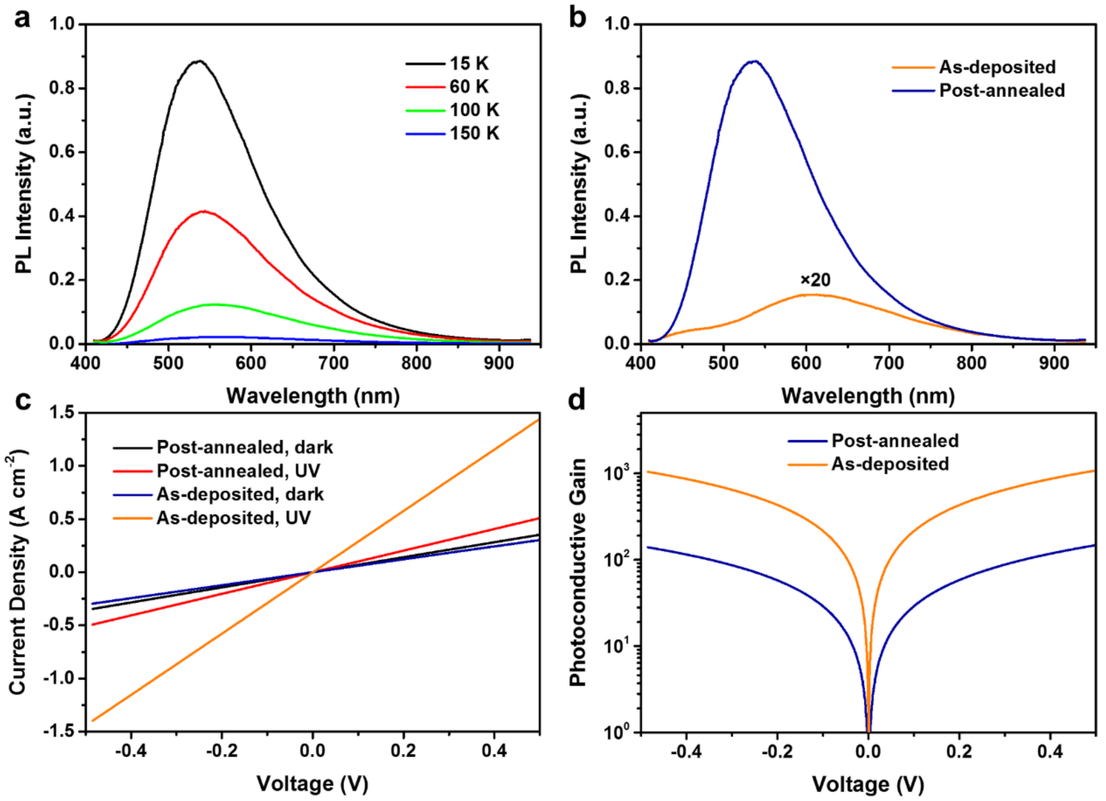

Supplementary Figure 13 | Optical and optoelectronic properties of post-annealed TiO_2 thin films. (**a**) Low temperature PL of a post-annealed TiO_2 thin film on a quartz substrate under 350 nm laser excitation. (**b**) Comparison of the PL emission for as-deposited and post-annealed TiO_2 thin films. The blue shift of the PL peak indicates that the distribution of defect states within the TiO_2 bandgap is altered by annealing and the greatly enhanced PL intensity suggests that non-radiative recombination channels are reduced. However, we note that the weak PL is consistent with dominant recombination via non-radiative processes in both cases. (**c**) Comparison of the conductivity for as-deposited and post-annealed TiO_2 thin films in dark and under AM 1.5G illumination. The dark conductivity was slightly enhanced after annealing. However, the photoconductivity was drastically decreased due to reduced hole-trapping at long-lived deep level states. (**d**) Photoconductive gain for as-deposited and post-annealed TiO_2 thin films.

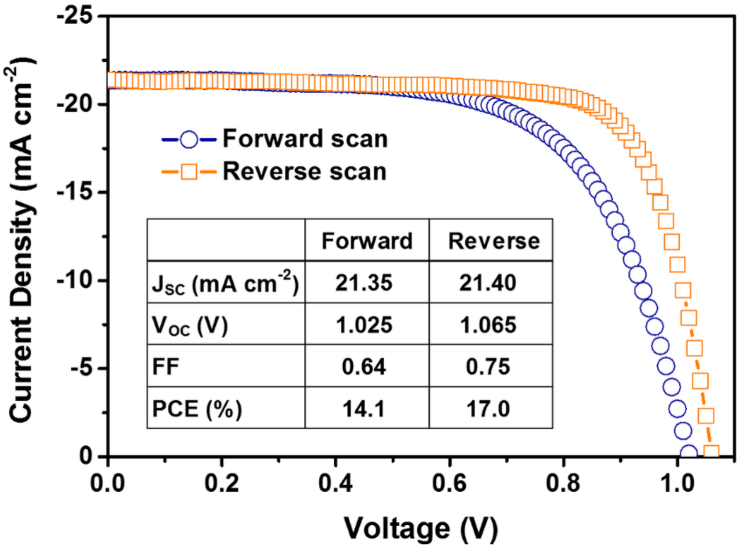

Supplementary Figure 14 | *JV* curves of a representative PSC fabricated using a post-annealed TiO_2 thin film as the ETL. The curves measured under forward and reverse scan at a rate of 10 mV per step under 100 mW cm^{-2} AM 1.5G illumination.

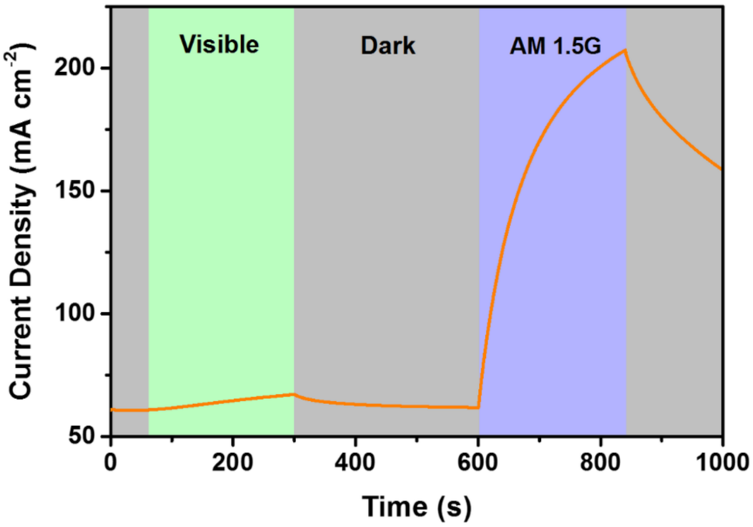

Supplementary Figure 15 | Photoresponse of the as-deposited TiO_2 thin film photoconductor under visible and AM 1.5G simulated sunlight illumination. Visible illumination was obtained by using a 425-nm long pass filter to block the UV light in the simulated sunlight.

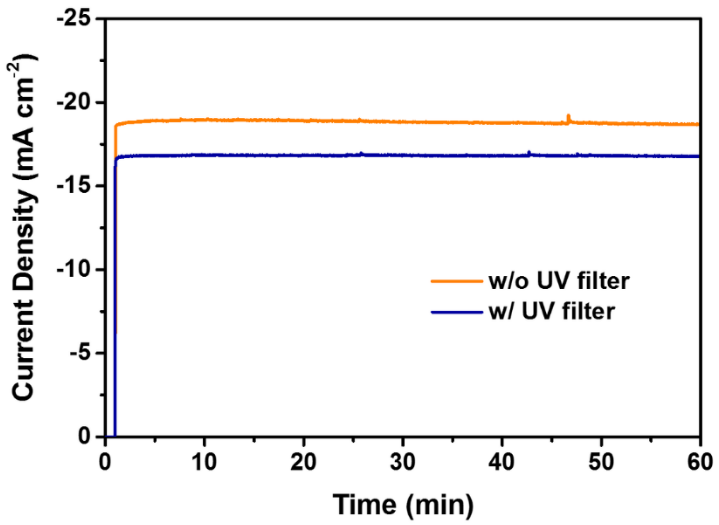

Supplementary Figure 16 | Photocurrent of the planar heterojunction PSC fabricated using post-annealed TiO_2 thin film as the ETL with (orange curve) and without (blue curve) UV illumination. The curves were measured at a bias of 0.8 V and a 425-nm long pass filter was used to block the UV light in the simulated sunlight. The devices were not subjected to any illumination before the test.

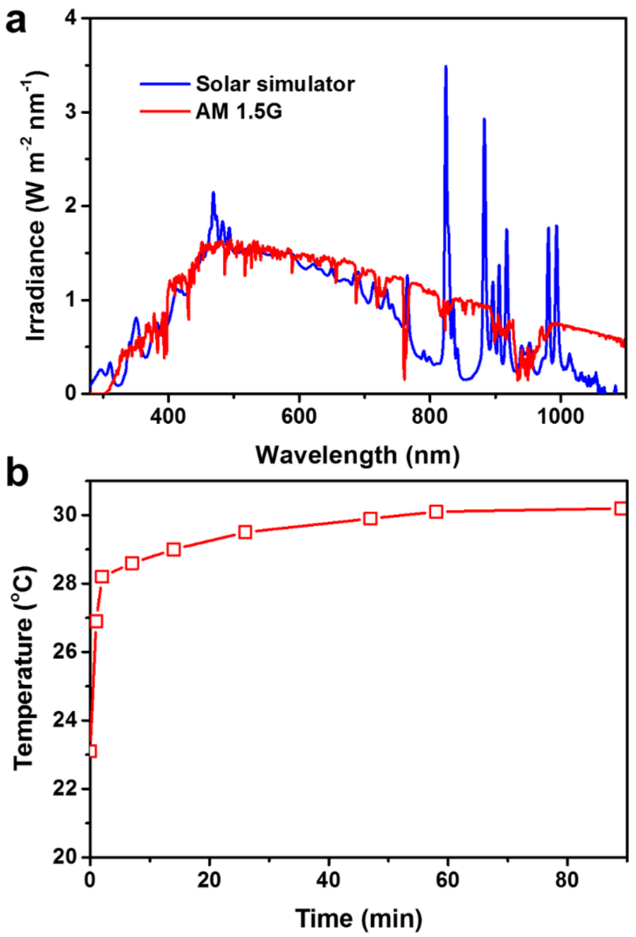

Supplementary Figure 17 | Measurement condition for the long term stability test. (**a**) Spectrum of the solar simulator used for long term stability. (**b**) Light-induced temperature increase during long term stability test.

Supplementary Table 1 | Photovoltaic performance of the planar heterojunction PSCs fabricated using post-annealed TiO_2 thin film as the ETL. Statistics are given for 10 devices with mean values and standard deviations for the solar cell parameters.

Scan direction	J_{SC} (mA cm^{-2})	V_{OC} (V)	*FF*	*PCE* (%)
Reverse	21.6±0.4	1.05±0.01	0.74±0.02	16.8±0.5
Forward	21.5±0.4	1.01±0.03	0.64±0.02	13.9±0.5
